# Supplementary material for: Liver-Specific Activation of AMPK Prevents Steatosis on a High-Fructose Diet
Source: Cell Rep. 2017 Mar 28;18(13):3043–51. doi: 10.1016/j.celrep.2017.03.011 (PMC5382239; doi:10.1016/j.celrep.2017.03.011)
Supplement: Document S1. Supplemental Experimental Procedures and Figure S1 [file mmc1.pdf]

Cell Reports, Volume *18*

Supplemental Information

Liver-Specific Activation of AMPK Prevents Steatosis on a High-Fructose Diet

Angela Woods, Jennet R. Williams, Phillip J. Muckett, Faith V. Mayer, Maria Liljevald, Mohammad Bohlooly-Y, and David Carling

Figure S1. Related to Figure 4

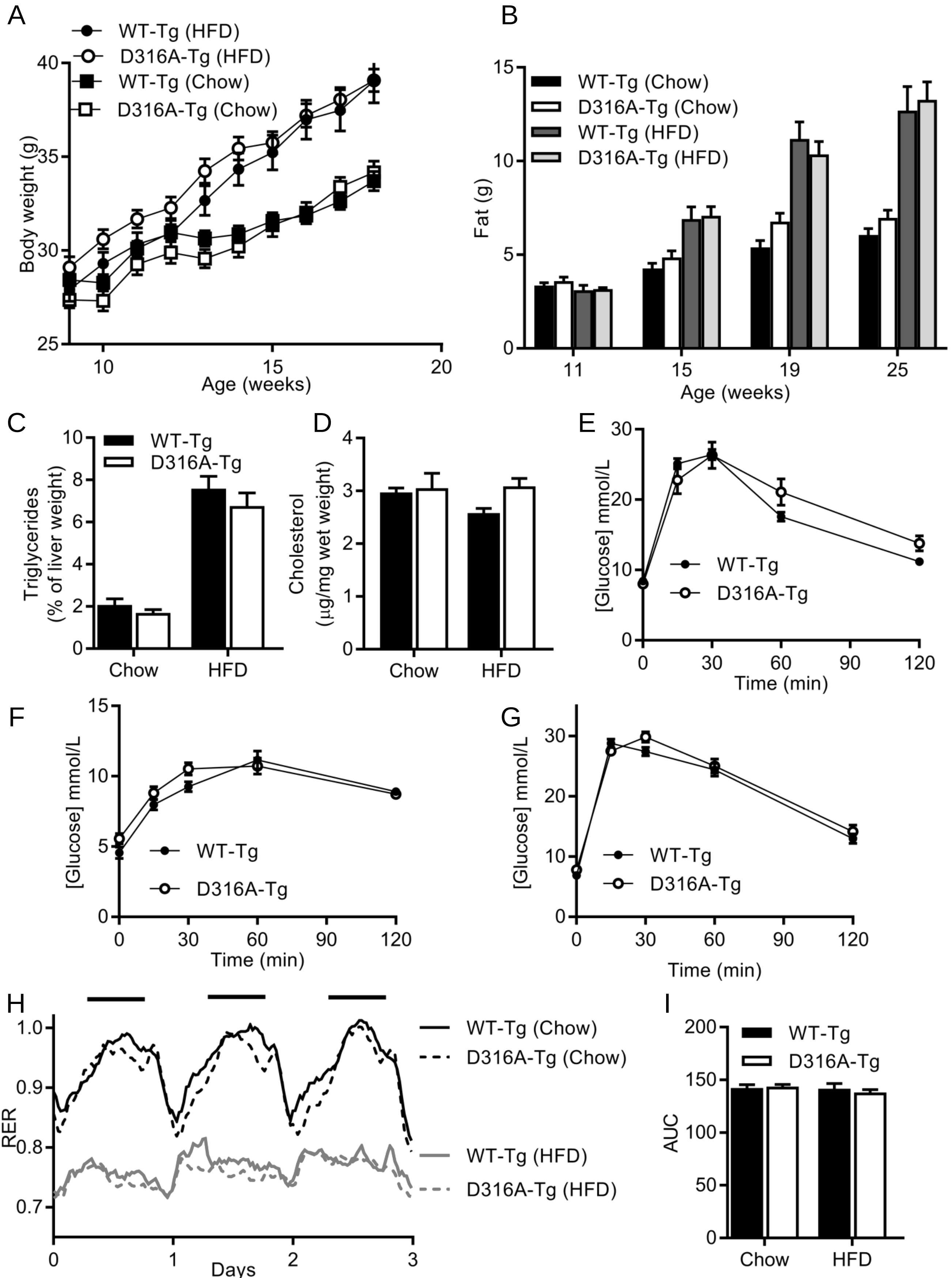

Figure S1. Metabolic effects of chronic hepatic activation of AMPK on mice fed a standard chow or high fat diet.

(A) Body weights of transgenic mice expressing either wild type γ1 AMPK (WT-Tg) or D316Aγ1 (D316A-Tg) fed a chow diet or a high fat diet (HFD) from 8 weeks of age. Results shown are the mean ± S.E.M. (in each case, n=24). (B) Total body fat, as assessed by EchoMRI (n=13-24). (C) Liver triglyceride and (D) liver cholesterol content (n= 9-12). (E) Glucose and (F) pyruvate tolerance tests of mice maintained on chow diet, and (G) glucose tolerance of mice fed a high fat diet for 12 weeks (n=9-10 per group). (H) Respiratory exchange ratio (RER) measured over 3 days for mice maintained on chow diet, or fed a high fat diet for 12 weeks (solid black bars represent hours of dark 19.00-7.00). (I) Total area under the curve (AUC) for VO_2 measured for mice maintained on chow diet, or fed a high fat diet for 12 weeks (n=14). In all cases results are shown as mean ± S.E.M. for the indicated number of mice.

Supplemental Experimental Procedures

Animal models

The *Rosa26* gene targeting vector was prepared from a mouse C57BL/6 bacterial artificial chromosome with homology arms 5.6 kb and 1.7 kb flanking the XbaI site in the *Rosa26* gene, described in detail previously (Soriano, 1999). A sequence encoding the Flag epitope (DYKDDDDK) at the C-terminus was engineered into the constructs to allow recognition by an anti-Flag antibody. Targeting vectors were linearized, and electroporated into PRXB6N mouse embryonic stem (ES) cells (Primogenix, Lauire, USA). Targeted ES cells were injected into BalbC/cANnCrl (Charles River, Germany) blastocysts and embryos were implanted into pseudo pregnant C57Bl6NCrl (Charles River, Germany) female mice. The resulting chimeric animals were mated with C57BL/6N mice (Charles River, Germany) to produce agouti heterozygous animals (F1). To generate animals without the Neo cassette, F1 mice were bred with CAG-FlpO. For genotyping the following primers were used: P1:MLi713F, accatgttcatgccttcttctt end of CAG promoter, P2=MLi748R, aattaggtcatagcagcgatga start of cDNA, detecting a fragment of 1.1 kb. These mice were crossed with *B6.Cg-Tg (Alb-Cre) 21 Mgn/J* transgenic mice harbouring Cre-recombinase under the albumin promoter (stock number 003574, Jackson Laboratories, Maine USA). Mice were maintained on a 12 h light/dark cycle with free access to food and water and housed in specific-pathogen free barrier facilities. Animals were killed by cervical dislocation and organs harvested rapidly and frozen in liquid nitrogen.

Primary mouse hepatocytes

Hepatocytes were isolated by collagenase perfusion of the liver of anesthetised mice. After isolation cells were seeded in collagen coated dishes in Medium 199 with Earles and L-glutamine (Gibco) supplemented with UltroserG (Pall Life Sciences), 1% albumin, 100 nM insulin, 100 nM triiodothyronine (T_3) and 100 nM dexamethasone. After cell attachment, the hepatocytes were cultured for 16–18 h in the absence of T3, albumin and UltroserG and in the presence of 1 nM insulin.

Hepatic Glucose Output

Cells were transferred into Dulbecco's Modified Eagles media without phenol red and supplemented with 2 mM sodium pyruvate and 20 mM lactate. Aliquots of media were removed from the cells and the glucose concentration measured using a glucose oxidase kit (Sigma).

Lipogenesis

Hepatocytes were incubated in M199 media containing 2 mM sodium acetate and 3 µCi/ml [1-^{14}C]sodium acetate (Perkin Elmer) for 4 h. Cells were washed thoroughly then scraped into methanol. Methanol was evaporated off and the pellet extracted with methyl-tert butyl ether:methanol (3:1), insoluble material was removed by centrifugation and the soluble fraction evaporated to dryness before determining ^{14}C incorporation by scintillation counting.

Fatty acid oxidation

Hepatocytes were incubated in M199 media containing 0.5 mM palmitate conjugated to BSA (2% w/v) and 1 µCi/ml [1-^{14}C]palmitic acid (Perkin Elmer). After 3-6 h media was removed and acidified with 0.1 volume 17 N acetic acid in an air-tight vial and the resulting CO_2 released was trapped in filter paper soaked in 1 M NaOH. The ^{14}C dissolved in NaOH was quantified by scintillation counting. Cells were washed thoroughly in PBS, extracted with chloroform/methanol and ^{14}C incorporated into aqueous soluble intermediates was determined. Total oxidation was calculated as the sum of $^{14}CO_2$-produced and ^{14}C incorporated into incomplete oxidation products.

Quantitative RT-PCR analysis

RNA was isolated from livers by homogenization in Trizol reagent (Invitrogen) according to the manufacturer's instructions, followed by purification on an RNeasy column (Qiagen). First strand cDNA synthesis using Superscript II (Invitrogen) according to the manufacturer's instructions and quantitative PCR was done with SensiMix Plus SYBR kit (Quantace) using the following primers:

G6Pase: CGACTCGCTATCTCCAAGTGA, GTTGAACCAGTCTCCGACCA
PEPCK: CCCCTTGTCTATGAAGCCCTCA, GCCCTTGTGTTCTGCAGCAG
ACC1: ATGGGCGGAATGGTCTCTTTC, TGGGGACCTTGTCTTCATCAT
FAS: TGCTCCCAGCTGCAGGC, GCCCGGTAGCTCTGGGTGTA
SREBP1c: GGAGCCATGGATTGCACATT, GCTTCCAGAGAGGAGGCCAG
SPOT14: Primers were obtained from Qiagen (no sequence information provided).

AMPK assay

AMPK was immunoprecipitated from liver or hepatocyte cell lysates using either a rabbit pan-β antibody bound to protein A-Sepharose (Woods et al., 1996) or anti-Flag affinity resin (Sigma) and activity present in the immune complexes was determined by phosphorylation of the SAMS peptide, as previously described (Davies et al., 1989).

Western blotting

Proteins were resolved by SDS-PAGE (Novex bis-tris 4-12% gradient gels) and transferred to polyvinylidene difluoride membrane. Primary antibodies used were: actin (Sigma), FAS (BD laboratories), FGF21 (Sigma), PP2Cα (Acris Antibodies), SCD1 (Life Technology), tubulin (Sigma). ACC, pACC (S79), AMPKα1/α2, AMPKβ1/β2, pAMPK (T172) and Flag were all from Cell Signaling Technology. Primary antibodies were detected using LI-COR IRDye® Infrared Dye secondary antibodies and visualised using an Odyssey Infrared Imager (LI-COR Biotechnology). Quantification of results was performed using Odyssey software and in some cases expressed as a ratio of the signal obtained with the phospho-specific antibody relative to an appropriate loading control antibody.

Recombinant AMPK expression

AMPK complexes were expressed in *E.coli* BL21 (DE3) cells, purified by affinity chromatography using nickel-Sepharose and phosphorylated by incubation with CaMKKβ as described previously (Sanders et al., 2007). Dephosphorylation of AMPK by recombinant PP2Cα was monitored either by Western blotting of phospho-T172 or by assaying for AMPK activity using the phosphorylation of the SAMS peptide. AMP present in perchloric acid extracts of purified AMPK was determined by ion-exchange chromatography as previously described (Fryer et al., 2002). Prior to extraction with perchloric acid, ADP (4 nmol) was added to control for efficiency of the extraction process.

Metabolic parameters

In vivo oxygen consumption and carbon dioxide production were measured using a Columbus Instruments Comprehensive Lab Animal Monitoring System over a 3 day period and used to calculate respiratory exchange ratio. Serum levels of total cholesterol, LDL and HDL cholesterol, triglycerides, 3-hydroxybutyrate and liver transaminases were determined by the Mouse Biochemistry Laboratories, Cambridge, UK. Lipids were extracted from tissue by homogenisation in ethanol (0.03 x w/v). Triglyceride content was measured using Triglyceride liquid (Sentinel Diagnostics) and cholesterol measured using Amplex Red Cholesterol kit (Molecular Probes). Liver glycogen was determined by alkaline hydrolysis by incubating tissue in 30% KOH at 100°C for 1h. Glycogen was precipitated in the cold by addition of 10x volume of ethanol and 7 μM LiCl. Precipitated glycogen was collected by centrifugation and dissolved in 4 M HCl, incubated for 1h at 100°C and neutralised by addition of an equal volume of K_2CO_3. The amount of glycogen converted to glucose was determined using a glucose oxidase kit (Sigma). Glucose and pyruvate tolerance tests were performed on mice after a 16 hour overnight fast. Animals were injected intraperitoneally with glucose (2 g/kg or 1.5 g/kg for mice on high fat diet) or pyruvate (2 g/kg) and blood glucose levels determined by a glucometer at the indicated time points. Body composition for determining body fat was measured by MRI using an EchoMRI-100 (Echo Medical Systems).

Supplemental References

Davies, S.P., Carling, D., and Hardie, D.G. (1989). Tissue distribution of AMP-activated protein kinase, and lack of activation by cyclic AMP-dependent protein kinase, studied using a specific and sensitive peptide assay. Eur. J. Biochem. *186*, 123-128.

Fryer, L.G., Parbu-Patel, A., and Carling, D. (2002). The Anti-diabetic drugs rosiglitazone and metformin stimulate AMP-activated protein kinase through distinct signaling pathways. J. Biol. Chem. *277*, 25226-25232.

Sanders, M.J., Grondin, P.O., Hegarty, B.D., Snowden, M.A., and Carling, D. (2007). Investigating the mechanism for AMP activation of the AMP-activated protein kinase cascade. Biochem. J. *403*, 139-148.

Soriano, P. (1999). Generalized lacZ expression with the ROSA26 Cre reporter strain Nat. Genet. *21*, 70-71.

Woods, A., Cheung, P.C., Smith, F.C., Davison, M.D., Scott, J., Beri, R.K., and Carling, D. (1996). Characterization of AMP-activated protein kinase beta and gamma subunits. Assembly of the heterotrimeric complex in vitro. J. Biol. Chem. *271*, 10282-10290.
