# Supplementary material for: Liver-Specific Activation of AMPK Prevents Steatosis on a High-Fructose Diet
Source: Cell Rep. 2017 Mar 28;18(13):3043–51. doi: 10.1016/j.celrep.2017.03.011 (PMC5382239; doi:10.1016/j.celrep.2017.03.011)
Supplement: Document S2. Article plus Supplemental Information [file mmc2.pdf]

Report

Cell Reports

Liver-Specific Activation of AMPK Prevents Steatosis on a High-Fructose Diet

Graphical Abstract

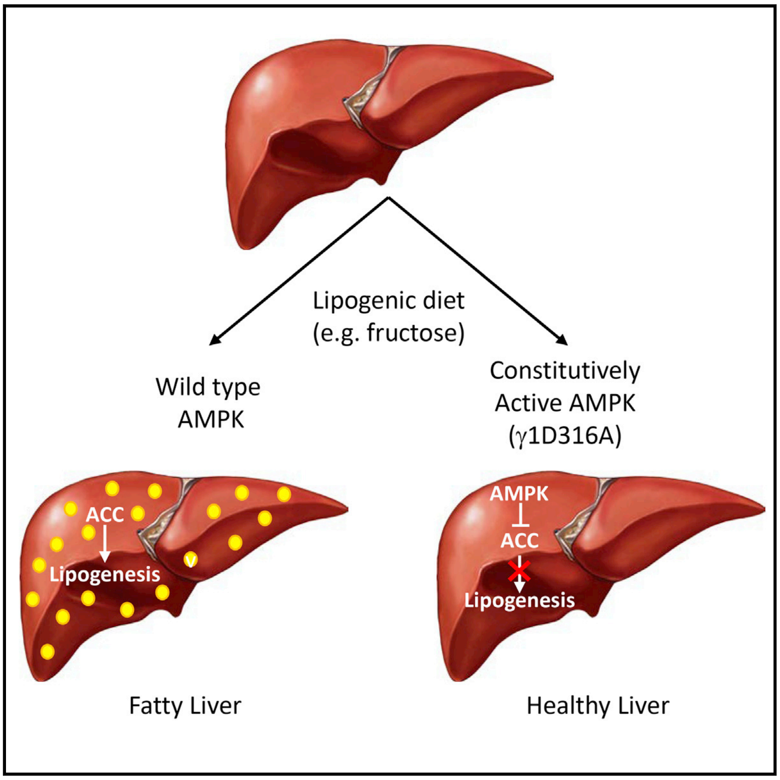

Highlights

- A mutation in AMPKγ1 subunit leads to constitutive activation of the kinase
- AMPK activation in liver protects against steatosis on a fructose diet
- AMPK inhibits de novo lipogenesis with no effect on hepatic fatty acid oxidation
- AMPK provides an attractive pharmacological target for protection against NAFLD

Authors

Angela Woods, Jennet R. Williams, Phillip J. Muckett, Faith V. Mayer, Maria Liljevald, Mohammad Bohlooly-Y, David Carling

Correspondence

angela.woods@lms.mrc.ac.uk (A.W.), david.carling@lms.mrc.ac.uk (D.C.)

In Brief

Using a gain-of-function mouse model, Woods et al. show that hepatic activation of AMPK protects against triglyceride accumulation in the liver. AMPK inhibits de novo lipogenesis but has no effect on hepatic fatty acid oxidation. These findings highlight AMPK as an attractive therapeutic target for protection against fatty liver disease.

Woods et al., 2017, Cell Reports *18*, 3043–3051
March 28, 2017 © 2017 The Author(s).
http://dx.doi.org/10.1016/j.celrep.2017.03.011

Cell Reports
Report

OPEN ACCESS
CellPress

Liver-Specific Activation of AMPK Prevents Steatosis on a High-Fructose Diet

Angela Woods,[1,*] Jennet R. Williams,[1] Phillip J. Muckett,[1] Faith V. Mayer,[1] Maria Liljevald,[3] Mohammad Bohlooly-Y,[4] and David Carling[1,2,5,*]
[1]MRC London Institute of Medical Sciences, Imperial College London, Hammersmith Hospital, London W12 0NN, UK
[2]Institute of Clinical Sciences, Imperial College London, Hammersmith Hospital, London W12 0NN, UK
[3]Drug Safety and Metabolism, Innovative Medicines and Early Development Biotech Unit, AstraZeneca, Pepparedsleden 1, Mölndal 431 83, Sweden
[4]Discovery Sciences, Innovative Medicines and Early Development Biotech Unit, AstraZeneca, Pepparedsleden 1, Mölndal 431 83, Sweden
[5]Lead Contact
*Correspondence: angela.woods@lms.mrc.ac.uk (A.W.), david.carling@lms.mrc.ac.uk (D.C.)
http://dx.doi.org/10.1016/j.celrep.2017.03.011

SUMMARY

AMP-activated protein kinase (AMPK) plays a key role in integrating metabolic pathways in response to energy demand. We identified a mutation in the γ1 subunit ($\gamma 1^{D316A}$) that leads to activation of AMPK. We generated mice with this mutation to study the effect of chronic liver-specific activation of AMPK in vivo. Primary hepatocytes isolated from these mice have reduced gluconeogenesis and fatty acid synthesis, but there is no effect on fatty acid oxidation compared to cells from wild-type mice. Liver-specific activation of AMPK decreases lipogenesis in vivo and completely protects against hepatic steatosis when mice are fed a high-fructose diet. Our findings demonstrate that liver-specific activation of AMPK is sufficient to protect against hepatic triglyceride accumulation, a hallmark of non-alcoholic fatty liver disease (NAFLD). These results emphasize the clinical relevance of activating AMPK in the liver to combat NAFLD and potentially other associated complications (e.g., cirrhosis and hepatocellular carcinoma).

INTRODUCTION

The incidence of non-alcoholic fatty liver disease (NAFLD) is increasing rapidly and is now one of the most common diseases worldwide. A recent meta-analysis study concluded that the global prevalence of NAFLD is over 25% (Younossi et al., 2016). While NAFLD can be benign, approximately 25% of patients with the disease will go on to develop non-alcoholic steatohepatitis (NASH), a more serious disease characterized by hepatic inflammation (Matteoni et al., 1999; Younossi et al., 2016). Patients with NASH risk developing additional complications, and 2% of patients will die of NAFLD liver-related mortality (Younossi et al., 2016). The initial hallmark of NAFLD is excessive accumulation of liver triglycerides (Postic and Girard, 2008). Currently, there are no approved drugs available for the treatment of NAFLD or NASH, and these diseases represent a major unmet clinical need.

AMP-activated protein kinase (AMPK) plays an important role in maintaining energy homeostasis (Carling et al., 2012; Steinberg and Kemp, 2009). Activation of AMPK leads to a decrease in ATP-consuming pathways and an increase in ATP-producing pathways. Given its key role in regulating energy balance, AMPK has become an attractive target for treatments aimed at diseases caused by disruption of energy metabolism. A number of direct AMPK activators have been reported (Cool et al., 2006; Xiao et al., 2013; Zadra et al., 2014), although, to date, the poor bioavailability of the compounds has limited their use in vivo. The first direct activator to be reported, a thienopyridone, A769662, was shown to reverse some of the metabolic disorders associated with the metabolic syndrome (Cool et al., 2006). A number of compounds lead to activation of AMPK through indirect mechanisms, including the widely used antidiabetic drug, metformin (Dandapani and Hardie, 2013). A caveat with interpreting the effects of pharmacological studies is the possibility of off-target effects of drugs. Another potential weakness is that it can be difficult to determine the particular cell type(s) involved in response to the drug.

A number of studies have examined the effects of specific AMPK subunits in vivo using transgenic knockout mouse models (Viollet et al., 2009). These studies, however, do not address the effect of AMPK activation on whole-animal physiology. Naturally occurring gain-of-function mutations in the γ2 subunit of AMPK have been identified in humans with heart disease (Arad et al., 2007) and in the γ3 subunit in Hampshire pigs with skeletal muscle glycogen storage disease (Milan et al., 2000). However, the limited tissue distribution of γ2 (predominantly expressed in heart) and γ3 (predominantly expressed in skeletal muscle) limits their suitability for use as models to understand the role of AMPK in non-muscle tissues. In an attempt to overcome these limitations, we identified a gain-of-function mutation in the widely expressed γ1 subunit of AMPK. Transgenic mice expressing this mutant form of γ1 in a conditional manner were generated and used to investigate the effects of liver-specific activation of AMPK.

This is an open access article under the CC BY license (http://creativecommons.org/licenses/by/4.0/).

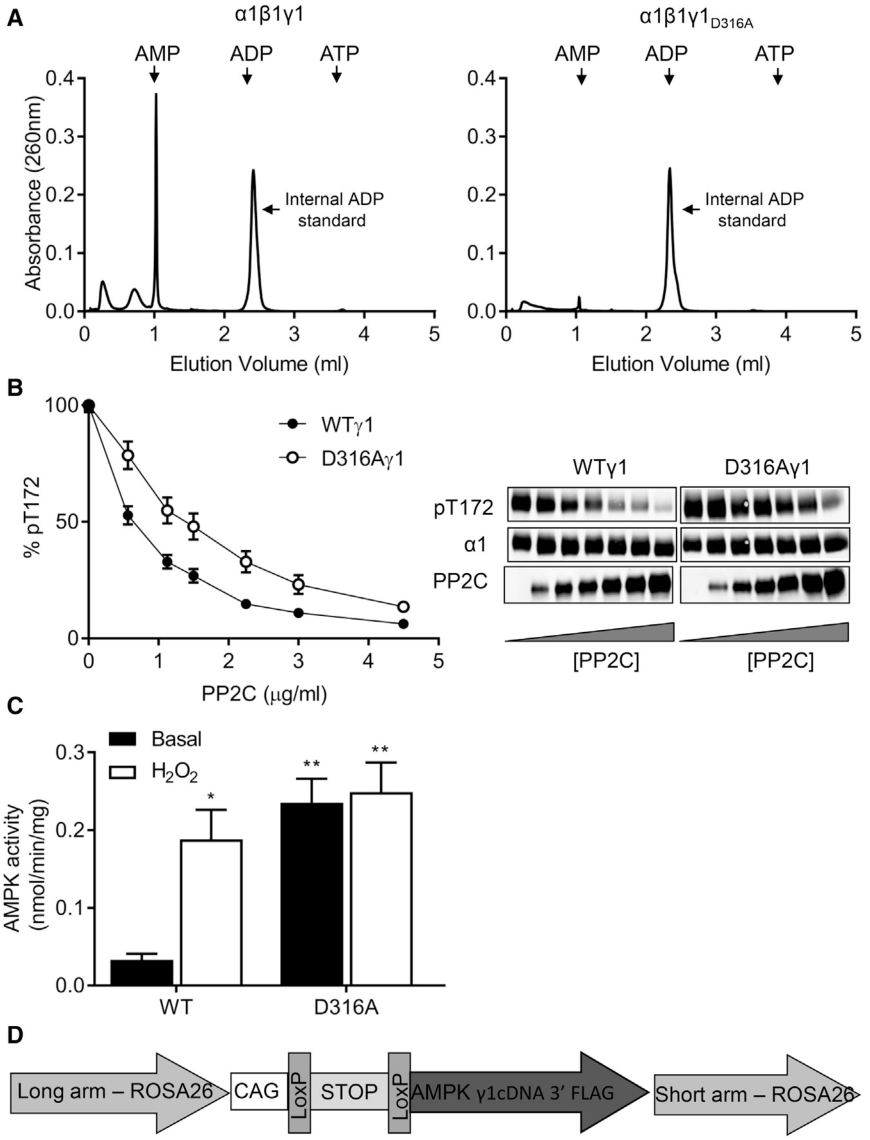

Figure 1. Characterization of γ1D316A Mutation In Vitro

(A) AMP in perchloric acid extracts of bacterially expressed AMPK complexes (wild-type $\alpha1\beta1\gamma1$ or $\alpha1\beta1\gamma1_{D316A}$) was determined by ion-exchange chromatography. The elution positions of AMP, ADP, and ATP standards are indicated by the arrows. ADP (4 nmol) was added prior to extraction as an internal standard and is marked by an arrow.

(B) Following phosphorylation by CaMKKβ, recombinant AMPK complexes were incubated in the presence of increasing concentrations of Protein phosphatase 2C (PP2C) for 20 min and then analyzed for T172 phosphorylation by western blotting. A representative blot showing the level of T172 phosphorylation, total AMPKα1, and PP2C is shown. Quantification of T172 phosphorylation relative to a control (in the absence of PP2C) is shown (n = 4).

(C) COS7 cells were co-transfected with cDNAs encoding α1, β1, and either wild-type or D316A γ1, harboring a C-terminal Flag epitope tag. AMPK activity from cells treated with or without 1 mM H_2O_2 for 15 min was measured in immune-complexes isolated with anti-Flag antibody (n = 4, *$p < 0.05$, **$p < 0.01$).

(D) Schematic representation of the construct used to generate transgenic mice expressing either WTγ1 or D316Aγ1.

Here, we show that liver-specific activation of AMPK has no significant metabolic effect on mice fed a normal chow diet or a high-fat diet. Activation of hepatic AMPK decreases de novo lipogenesis, and this protects against the development of liver steatosis following a high-fructose diet. Our findings have important implications for understanding the impact of potential therapeutic targeting of AMPK. By reducing de novo lipogenesis, activation of hepatic AMPK would be anticipated to slow the progression of NAFLD, decreasing the risk of associated complications, such as NASH, cirrhosis, and hepatocellular carcinoma (Matteoni et al., 1999).

RESULTS

During our previous analysis of adenine nucleotide binding to AMPK, we identified aspartic acid residues present within each of the three occupied sites in the γ1 subunit that interact with the 2′ and 3′ hydroxyl groups of the ribose moiety (Xiao et al., 2007). The tightly bound AMP in site 4, which we found to be non-exchangeable in solution, interacts with aspartic acid residue 316 (residue 317 in human γ1). Mutation of D316 to an alanine (D316A) abolished binding of this tightly bound AMP molecule (Figure 1A) and rendered the complex less sensitive to dephosphorylation by protein phosphatase 2C (PP2C) (Figure 1B). In mammalian cells, co-expression of γ1 harboring the D316A mutation with α1 and β1 led to increased basal AMPK activity compared to wild-type enzyme (Figure 1C). Wild-type AMPK was activated approximately 7-fold by treatment of the cells, with hydrogen peroxide bringing its activity to a similar level as the D316A mutant. Hydrogen peroxide treatment had no significant effect on the activity of the D316A mutant AMPK complex. These results show that mutation of D316 to alanine in γ1 leads to a gain of function in AMPK activity, presumably mediated by a decrease in the rate of dephosphorylation of T172.

We designed Cre/loxP conditional ROSA26 targeting vectors for expression of either wild-type or mutant γ1 and used these to generate transgenic mouse lines (hereafter referred to as wild-type γ1 [WT-Tg] or D316A mutant γ1 [D316A-Tg] transgenic mice). We engineered a sequence encoding the Flag epitope at the C terminus to allow recognition by an anti-Flag antibody (see Figure 1D). To generate liver-specific expression of the

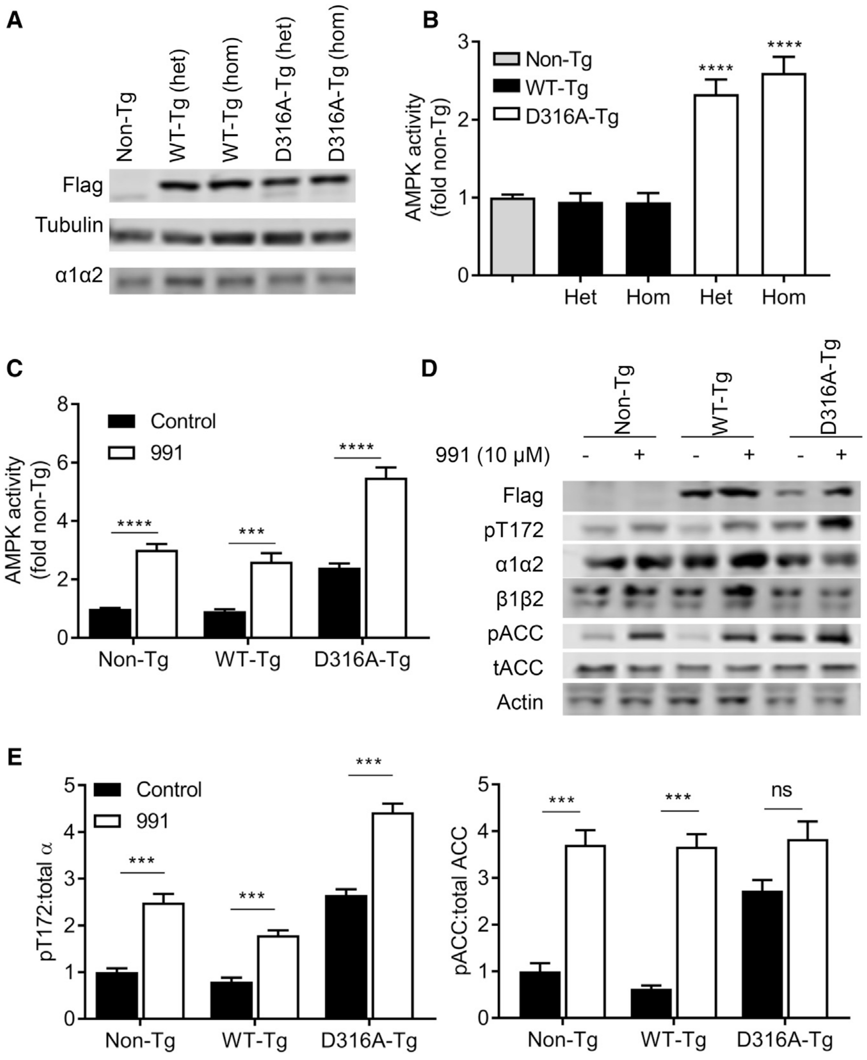

Figure 2. Expression of γ1D316A in Mouse Hepatocytes

(A) Hepatocyte lysates isolated from mice either heterozygous (het) or homozygous (hom) for WT or D316A γ1 transgene were blotted with the indicated antibodies.

(B) AMPK activity was measured in immune complexes isolated with a pan AMPKβ antibody. Results shown are plotted as fold activity relative to hepatocytes from Non-Tg mice.

(C) Hepatocytes were treated in the presence or absence of 10 μM 991 for 30 min, and AMPK activity was measured in immune complexes isolated with a pan AMPKβ antibody. Results shown are plotted relative to the activity in Non-Tg, untreated control cells, from at least six independent experiments.

(D and E) (D) A representative blot of hepatocyte lysates after treatment with or without 10 μM 991 for 30 min probed with the indicated antibodies and (E) quantification of blots for three independent experiments; ***$p < 0.005$, ****$p < 0.001$; NS, not significant.

transgenes, animals were crossed with mice expressing Cre-recombinase under the control of the albumin promoter (Postic et al., 1999). Measurement of AMPK activity in the liver can be confounded by post-mortem changes in T172 phosphorylation caused by rapid depletion of ATP during tissue isolation (Davies et al., 1992). In order to overcome this issue, we isolated primary hepatocytes to allow determination of AMPK activity in the basal state. Expression of both WT and D316A γ1 protein was confirmed by western blot analysis (Figure 2A). Similar levels of expression and AMPK activity (Figure 2B) were observed in mice either heterozygous or homozygous for the transgene. Importantly, however, under basal conditions, total AMPK activity was approximately 2.5-fold higher in hepatocytes from D316A-Tg mice compared to cells from either non-Tg or WT-Tg mice (Figures 2B and 2C). Following incubation with 991, a direct activator of AMPK (Xiao et al., 2013), AMPK activity was increased 2- to 3-fold in all cases (Figure 2C). Phosphorylation of T172 and acetyl-CoA carboxylase (ACC), a downstream substrate of AMPK, increased in parallel with the increase in AMPK activity (Figures 2D and 2E). These results demonstrate that in vivo expression of D316A γ1 increases basal AMPK activity to a similar level as that seen following pharmacological activation with a direct AMPK activator. No difference in total AMPK expression was detected in cells from Non-Tg, WT-Tg, or D316A-Tg mice, as assessed by blotting for either total α or β subunits (Figure 2D). We were unable to determine total γ1 expression, as the anti-γ1 antibody that we used was raised against the C-terminal region of γ1 and did not cross-react with the transgenic γ1 protein, presumably due to interference by the addition of the Flag epitope tag (data not shown). As there was no difference in AMPK activity in WT-Tg compared to Non-Tg hepatocytes, we used WT-Tg animals as controls for the remaining studies. Similarly, since we detected no difference in AMPK expression or activity between heterozygous or homozygous transgenic mice, we combined both heterozygous and homozygous mice for all further studies in order to maximize animal usage.

AMPK has been implicated in the regulation of a number of metabolic pathways in the liver, including gluconeogenesis, fatty acid synthesis, and fatty acid oxidation (FAO) (Hardie and Carling, 1997; Steinberg and Kemp, 2009). Basal and glucagon-stimulated glucose outputs were decreased by approximately 50% in hepatocytes isolated from D316A-Tg compared to WT-Tg cells (Figure 3A). Consistent with this effect, we found that expressions of glucose-6-phosphatase (G6Pase) and phosphoenolpyruvate carboxykinase (PEPCK) mRNA, genes coding for two key enzymes in gluconeogenesis, were significantly decreased in hepatocytes from the D316A-Tg mice (Figure 3B). Fatty acid synthesis, as measured by incorporation of ^{14}C-acetate into fatty acid, was significantly decreased in hepatocytes

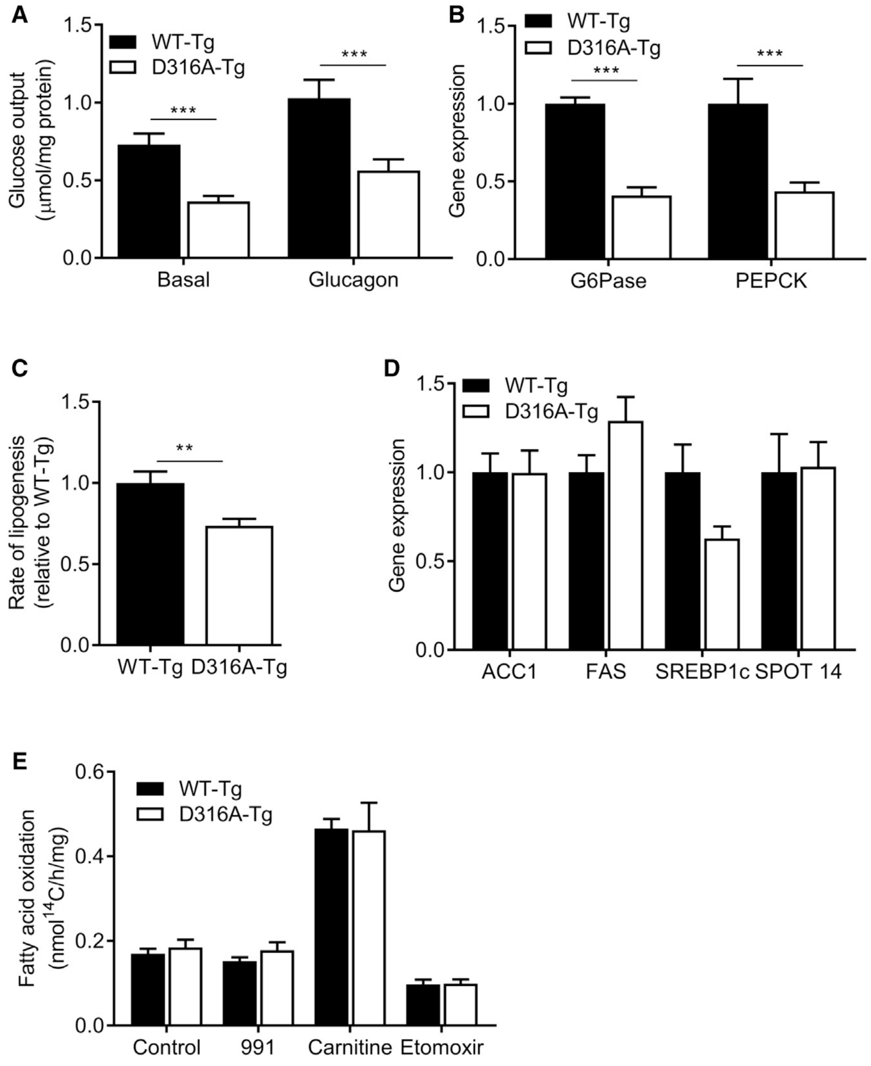

Figure 3. Metabolic Effects of Chronic Expression of γ1D316A in Mouse Hepatocytes

(A) Hepatocytes from transgenic mice were incubated in the presence or absence of 10 nM glucagon, and hepatic glucose output over 18 hr was measured and normalized to total cellular protein.

(B) Relative expression of gluconeogenic genes, glucose-6-phosphatase (G6Pase), and phosphoenolpyruvate carboxykinase (PEPCK).

(C) De novo lipogenesis was measured in isolated hepatocytes by incorporation of ^{14}C-acetate into lipids.

(D) Lipogenic gene expression plotted relative to expression in WT-Tg cells.

(E) Fatty acid oxidation was measured over 6 hr using ^{14}C-palmitate in the presence or absence of 10 μM 991, 1 mM carnitine, or 0.1 mM etomoxir. In each case, results are from at least six independent experiments; *p < 0.05, **p < 0.01, ***p < 0.005, ****p < 0.001.

from D316A-Tg compared to WT-Tg mice (Figure 3C), although we did not detect any significant change in expression of a number of genes involved in lipogenesis (Figure 3D). A number of studies have reported that treatments that cause activation of AMPK increase FAO. AMPK phosphorylates and inactivates ACC, which leads to reduced levels of malonyl-CoA, thereby increasing mitochondrial fatty acid uptake, increasing FAO. However, most of the previous reports linking AMPK activation to increased FAO have studied skeletal muscle, and very few studies have provided direct evidence that AMPK activation increases FAO in the liver (Fullerton et al., 2013). We did not detect any significant difference in fatty acid oxidation of ^{14}C-labeled palmitate in D316A-Tg cells relative to WT-Tg cells (Figure 3E). We also failed to detect a change in FAO following treatment with 991 (Figure 3E). Addition of carnitine increased, whereas treatment of the cells with etomoxir, an inhibitor of carnitine palmitoyl transferase I, reduced FAO, demonstrating that the hepatocytes have the capacity to respond to changes in FAO rates.

In order to determine the effect of liver-specific AMPK activation on whole-body metabolism in vivo, we measured a number of metabolic parameters in WT-Tg and D316A-Tg mice fed on a chow diet. We were unable to detect any significant differences in body weight, total body fat, liver triglycerides, liver cholesterol, glucose handling, respiratory exchange ratio (RER), or VO_2 consumption between the two genotypes (Figure S1). Similarly, we did not see any significant difference in these parameters when mice were fed a high-fat diet (Figure S1). No difference in RER was observed between WT-Tg and D316A-Tg mice on either a chow or high-fat diet. Consistent with previously published studies, RER values were lower in mice fed a high-fat diet, reflecting an increased reliance on FAO (Figure S1H). In light of our finding in isolated primary hepatocytes that AMPK activation had no effect on fatty acid oxidation, but did significantly reduce lipogenesis, we measured in vivo de novo lipogenesis rates. De novo lipogenesis was reduced by approximately 30% in D316A-Tg mice compared to WT-Tg mice (Figure 4A). In order to determine the physiological relevance of this effect, we fed mice a diet high in fructose (60% of calories derived from fructose), which is known to increase hepatic lipogenesis in rodents and humans (Mayes, 1993; Tappy and Lê, 2010). There was no significant difference in body weight, total fat mass, glucose tolerance, serum lipids, serum 3-hydroxybutyrate, RER, or VO_2 consumption between the WT-Tg and D316A-Tg mice after 12 weeks on the high-fructose diet (Figure 4). In contrast, there was a dramatic reduction in hepatic triglyceride content in liver from D316A-Tg mice compared to WT-Tg mice (Figure 5A). Remarkably, hepatic triglyceride levels in the D316A-Tg mice were similar to those in mice maintained

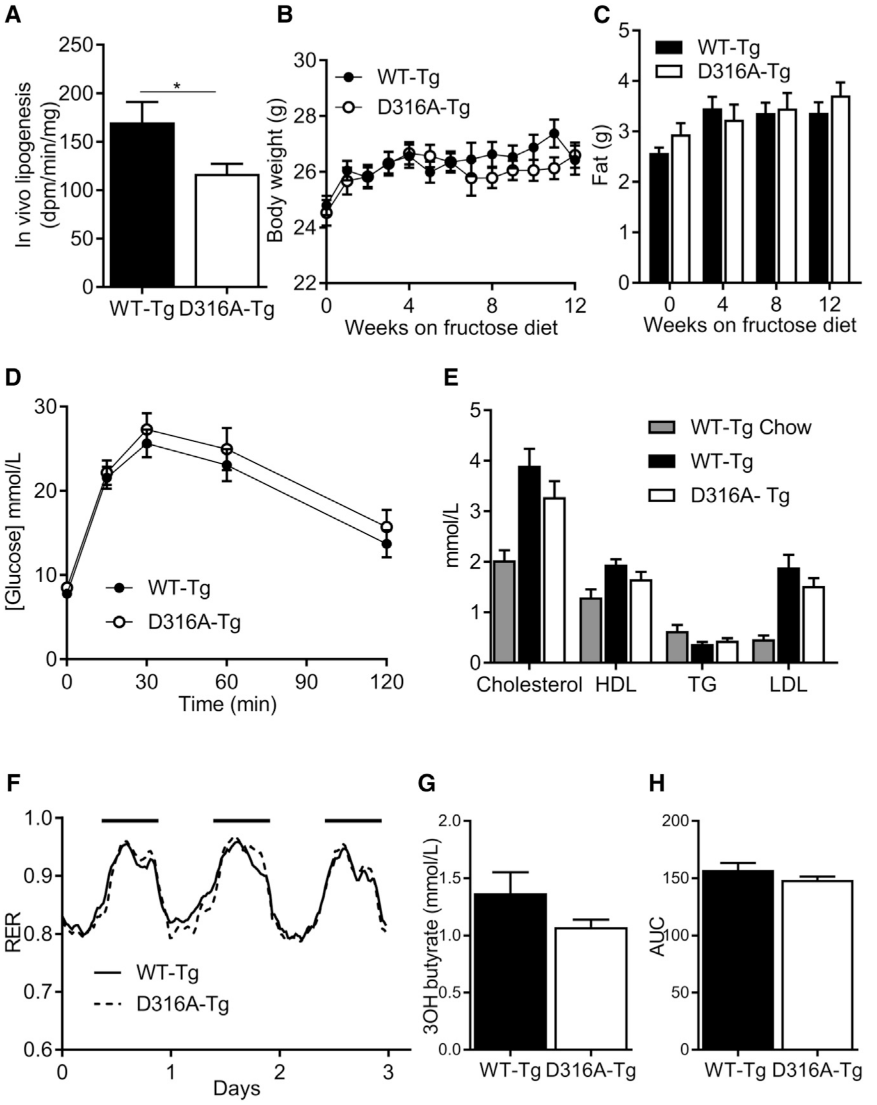

Figure 4. Whole-Body Metabolic Effects of Chronic Hepatic Activation of AMPK on Mice Fed a High-Fructose Diet

(A) In vivo hepatic lipogenesis was determined by measuring incorporation of ^{3}H-acetate into lipids (n = 8 per group).

(B) Body weights of mice fed a high-fructose diet from eight weeks of age (n = 20).

(C and D) (C) Total body fat measured by EchoMRI and (D) glucose tolerance test of mice fed a high-fructose diet for 12 weeks (n = 8–13).

(E) Serum lipids of mice fed a high-fructose diet for 12 weeks and compared to WT-Tg mice fed on chow (n = 9–12).

(F) Respiratory exchange ratio (RER) measured over 3 days for WT-Tg and D316A-Tg mice fed a high-fructose diet for 4 weeks. The solid black bars indicate the dark period between 19.00 and 07.00.

(G) Serum 3-hydroxybutyrate levels in mice fed a high-fructose diet for 12 weeks (n = 9–10).

(H) Total area under the curve (AUC) for VO_2 consumption measured over three days for mice fed a high-fructose diet for four weeks (n = 10–12).

on a normal chow diet (Figure 5A). No significant differences in liver cholesterol (Figure 5B), glycogen (Figure 5C), or serum transaminases (Figures 5D and 5E) were detected either between the different transgenic mice or between fructose and chow diets. Similarly, we did not detect any changes in expression of a number of genes correlated with liver injury, e.g., CCL2, CCL4, IL6, and TGFβ1 (data not shown). The expression of the lipogenic enzymes ACC, fatty acid synthase (FAS), and stearoyl-CoA desaturase (SCD1) were all increased in livers of mice fed a high-fructose diet relative to normal chow, and this was similar in both genotypes (Figures 5F and 5G). Expression of fibroblast growth factor 21 (FGF21) was also increased in response to the high-fructose diet in livers from both WT-Tg and D316A-Tg mice, consistent with previous studies showing increased expression in response to fructose (Dushay et al., 2014). These results indicate that the signaling pathway that leads to increased protein expression of lipogenic enzymes in response to fructose remains intact in the D316A-Tg mice. Haematoxylin and Eosin (H&E) staining of livers from WT-Tg mice fed on a high-fructose diet showed large lipid droplets, which were completely absent in livers from the D316A-Tg mice (Figure 5H).

DISCUSSION

Here, we describe a mouse model for investigating the effects of chronic AMPK activation in vivo. We found that mutation of aspartic acid residue 316 to alanine in mouse γ1 (residue 317 in human γ1) makes AMPK a worse substrate for dephosphorylation, leading to increased AMPK activity when expressed in mammalian cells. Generation of transgenic mice with conditional expression of this mutant provided us with the opportunity to investigate the consequence of chronic, tissue-specific activation of AMPK.

The liver plays a key role in regulating whole-body energy metabolism (Rui, 2014), and previous studies suggest an important role for AMPK in coordinating changes in hepatic metabolism required for maintaining overall energy balance (Davies et al., 1992; Woods et al., 2000). An advantage of expressing the transgene in liver was that it allowed us to use primary hepatocytes to characterize the effect of the γ1 mutation on AMPK and downstream pathways at a cellular level. Expression of the transgenic γ1 protein has no effect on the total level of AMPK complex, indicating that transgenic γ1 protein competes with endogenous γ1. Importantly, basal AMPK activity is 2- to 3-fold higher in hepatocytes isolated from D316A-Tg mice, compared to either WT-Tg or Non-Tg mice. This degree of activation is similar to that obtained following treatment of

OPEN
ACCESS
CellPress

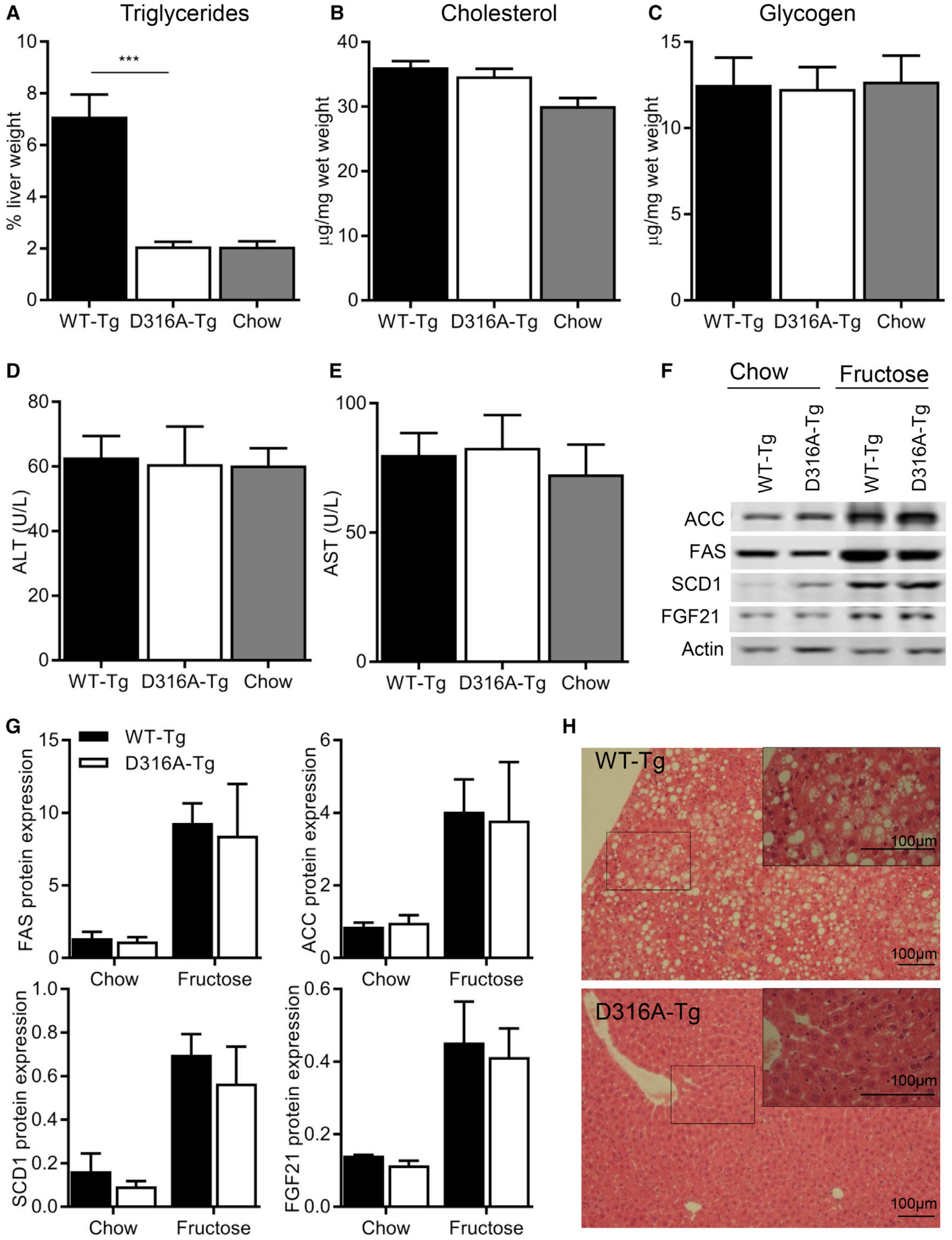

Figure 5. Effects of Liver-Specific Activation of AMPK on Hepatic Lipid Accumulation on a High-Fructose Diet

(A–C) (A) Triglyceride, (B) cholesterol, and (C) glycogen content of livers from transgenic mice fed a high-fructose diet for 12 weeks was determined (n = 12 mice, ***$p < 0.005$). Levels in livers from WT-Tg mice fed a chow diet are shown for comparison.

(D and E) Liver transaminase levels in serum (n = 10–12).

(F) A representative western blot of liver homogenates from mice fed either a chow or high-fructose diet probed with the indicated antibodies is shown.

(G) Quantification of the blots normalized to the level of actin expression (n = 4).

(H) Liver sections from transgenic mice fed a high-fructose diet for 12 weeks stained with H&E. The boxed area is shown magnified in the inset in the top right-hand corner of the image.

hepatocytes with the AMPK activator 991. This finding makes this genetic model an attractive one for understanding the likely pharmacological effects of liver-specific AMPK activation in vivo.

Previous studies have reported that pharmacological activation of AMPK inhibits hepatic gluconeogenesis (Bergeron et al., 2001; Zhou et al., 2001), although subsequent studies have shown that AMPK is not required for inhibition of gluconeogenesis by metformin (Foretz et al., 2010). Genetic activation of AMPK caused a significant reduction in glucose output in isolated hepatocytes, and this was likely mediated by decreased mRNA expression of G6Pase and PEPCK. While there are multiple pathways by which transcription of these two key gluconeogenic genes can be regulated, our results show that activation of AMPK per se has a significant effect on their expression and hepatic glucose output. However, despite the effect of AMPK activation on gluconeogenesis in isolated hepatocytes, we did not detect any change in glucose handling in vivo. This is not without precedent and could be explained by compensatory extrahepatic mechanisms. A previous study reported that mice lacking hepatic PEPCK expression, which have dramatically reduced hepatic glucose production, display near-normal blood glucose levels. This is achieved through compensatory mechanisms, including an increase in extrahepatic gluconeogenesis coupled with reduced whole-body glucose utilization (She et al., 2003).

Activation of AMPK in the liver has been reported to inhibit fatty acid synthesis and promote FAO via phosphorylation and inactivation of ACC. In parallel with increased phosphorylation of ACC, fatty acid synthesis was significantly reduced in hepatocytes expressing the D316A mutation. In contrast, there was no effect on FAO. Treatment of hepatocytes with 991 also had no effect on FAO. Fullerton et al. generated a mouse model in which the key AMPK phosphorylation sites in ACC1 (serine 79) and ACC2 (serine 212) were mutated to alanine residues (Fullerton et al., 2013). In that study, treatment of hepatocytes with A769662, a direct activator of AMPK, caused a modest increase in FAO in cells expressing wild-type ACC but had no effect in cells expressing the mutant ACC enzymes (Fullerton et al., 2013). Although we are unable to explain the difference between our results and those obtained in the previous study, it is interesting to note that, using the same ACC phosphorylation-deficient mouse model, phosphorylation of ACC by AMPK was not required for regulation of FAO in heart (Zordoky et al., 2014). In a more recent study, canagliflozin, which was shown to activate AMPK in the liver, decreased RER independently of ACC phosphorylation (Hawley et al., 2016). These findings suggest that the relationship between AMPK, ACC phosphorylation, and the regulation of FAO is not a simple one and that other mechanisms, independent of AMPK phosphorylation and inhibition of ACC, regulate FAO in vivo.

The effect of diet on hepatic fatty acid synthesis has been studied extensively in both rodents and humans. Increasing the proportion of carbohydrates in the diet leads to a marked increase in de novo lipogenesis in the liver, and this is associated with increased expression of enzymes involved in fatty acid synthesis (Girard et al., 1997). The effect of a high-fat diet is less clear, although a recent study in mice reported that a high-fat diet decreased de novo lipogenesis in the liver despite insulin resistance and obesity (Duarte et al., 2014). In healthy humans, it has been estimated that less than 5% of total triglyceride synthesis is accounted for by de novo lipogenesis on a moderate fat diet (30% fat and 55% carbohydrate), compared to over 25% on a low-fat diet (10% fat and 75% carbohydrate) (Hudgins et al., 2000). An increase in the consumption of sugars, including fructose, particularly in Western diets, has been linked to the increasing prevalence of obesity, together with associated metabolic diseases. The most striking effect of liver-specific AMPK activation that we observed is the complete protection against hepatic triglyceride accumulation in mice fed a high-fructose diet. The simplest explanation for this effect is that AMPK activation inhibits hepatic de novo fatty acid synthesis by directly inactivating ACC. Consistent with this, we found that de novo lipogenesis was reduced in isolated hepatocytes and in the liver in vivo in mice expressing the gain-of-function AMPK mutant. There was no obvious metabolic effect of AMPK activation in mice fed either a chow or a high-fat diet. Under these conditions, the rates of hepatic de novo lipogenesis will be low (relative to the rate on a high-fructose diet) and so would provide only a minor contribution toward total liver triglyceride accumulation. Importantly, activation of AMPK did not increase FAO in hepatocytes, and there was no difference in RER between WT-Tg and D316A-Tg mice on any diet. In addition, there was no change in serum 3-hydroxybutyrate levels between genotypes, indicating that FAO rates in vivo were not different. These results show that liver-specific activation of AMPK does not alter FAO rates in vivo. These findings suggest that the predominant metabolic effect of AMPK activation in the liver is suppression of de novo lipogenesis mediated by phosphorylation of ACC. We hypothesize that this occurs under all dietary conditions, and therefore a phenotype is only revealed under conditions leading to high rates of hepatic de novo lipogenesis (e.g., on a high-fructose diet).

A previous study reported beneficial effects of pharmacological activation of AMPK in animal models of the metabolic syndrome (Cool et al., 2006). In that study, acute treatment of rats in vivo with A769662 led to a transient decrease in RER, indicative of an increase in whole-body FAO. In the same study, chronic treatment of *ob/ob* mice with A769662 led to a small decrease in body weight, a decrease in fed plasma glucose levels, and a decrease in plasma and liver triglycerides. Although the authors speculated that these changes were likely to be mediated by activation of AMPK in the liver, other mechanisms, including AMPK activation in non-hepatic tissues, such as skeletal muscle, could not be ruled out (Cool et al., 2006). Another potentially confounding factor with the use of A769662 is that a subsequent study reported an AMPK-independent effect of A769662 in skeletal muscle cells (Benziane et al., 2009). Taking these considerations into account and in light of the results in our current study, it seems likely that at least some of the metabolic effects of A769662 treatment in vivo could be mediated independently of AMPK activation in the liver. A more recent study investigated the effect of chronic dosing with AICA (5 aminoimidazole-4-carboxamide) riboside in rats. Treatment with AICA riboside led to decreased hepatic triglyceride accumulation in rats maintained on either a chow or high-fat diet (Henriksen et al., 2013). As with the study using A769662 in vivo, it is not possible to determine the role of the liver-specific

activation of AMPK in these effects, since, as well as activating AMPK in other tissues, AICA riboside has been shown to have AMPK-independent effects.

In another study, short-term activation of AMPK in the liver was achieved by expression of a constitutively active truncated form of AMPKα2 using adenoviral delivery (Foretz et al., 2005). A number of changes were reported, including an increase in hepatic triglyceride accumulation, which the authors speculated could have been caused by increased mobilization of fatty acids from the adipose tissue. Whether this would still occur in response to chronic AMPK activation was not addressed. As well as differences in chronic versus acute activation, that study is confounded by the nature of the constitutively active AMPKα2 mutant used. Truncated α2 does not bind to the β and γ regulatory subunits (Crute et al., 1998; Stein et al., 2000), and so it is possible that this might alter the function of the mutant α2 subunit.

Previous studies have employed different genetic mouse models to determine the effect of AMPK activation in skeletal muscle (Barnes et al., 2004; Barré et al., 2007; Schönke et al., 2015). A striking feature of the mouse models is increased skeletal muscle glycogen accumulation, although no major changes in lipid metabolism were reported. A chronic gain-of-function AMPK model resulting from expression of AMPKγ2 with mutation of arginine 302 to glutamine (R302Q) was reported recently (Yavari et al., 2016). Mice with global homozygous expression of this mutation are hyperphagic, obese, and display impaired pancreatic insulin secretion (Yavari et al., 2016). At least some of these effects appear to be mediated by changes in AMPK activity in the hypothalamus. Whether these effects result from an overall increase in AMPK activity, or are due to a specific increase in γ2-associated AMPK activity, is unclear at present. Further studies using the gain-of-function AMPKγ1 mouse model should help address both the isoform- and tissue-specific effects of AMPK activation in vivo.

In summary, our study reveals that AMPK activation in liver completely prevents lipid accumulation on a high-carbohydrate diet. This finding may have implications for the therapeutic potential of targeting hepatic AMPK. The incidence of NAFLD is increasing rapidly and is associated with the increasing prevalence of obesity. Although NAFLD is classified as a benign condition, many individuals with the disease go on to develop more serious conditions, including NASH, cirrhosis, liver failure, and hepatocellular carcinoma. De novo lipogenesis is significantly increased in patients with NAFLD, and this increase is thought to contribute to the accumulation of hepatic triglycerides in NAFLD (Donnelly et al., 2005). Our findings indicate that direct activation of AMPK in the liver might provide an attractive therapeutic strategy for preventing progression of NAFLD and subsequent development of associated complications.

EXPERIMENTAL PROCEDURES

Animal Models

cDNA (Epoch Life Science) for the mouse *Prkag1* gene and cDNA harboring the D316A mutation were specifically integrated into the *Rosa26* locus. Chow-standard breeding diet number 3 was from Special Diets Services; high-fat diet (45% energy from fat) and high-fructose diet (60% fructose diet with 10% fat) were obtained from TestDiet. All experiments involved in the generation of transgenic animals were approved by Gothenburg Ethics Committee. All in vivo studies were performed in accordance with the UK Animals (Scientific Procedures) Act (1986) and approved by the Animal Welfare and Ethical Review Board at Imperial College London.

In Vivo Lipogenesis

De novo lipogenesis was determined by incorporation of ^{3}H-acetate into lipid. Mice were fasted overnight and refed for 1.5 hr before intraperitoneal injection with 40 μCi ^{3}H-acetate (Perkin Elmer). After 1 hr, mice were sacrificed and livers extracted using the Folch Method. Incorporation of radioactivity in the lipid fraction was determined by scintillation counting.

Histology

Tissue was fixed in 4% paraformaldehyde and processed to paraffin wax. Sections were stained with H&E.

Statistical Analysis

Data are expressed as mean ± SEM unless otherwise stated. Where appropriate, results were analyzed using either Student's t test or ANOVA with Dunnet's post-test.

SUPPLEMENTAL INFORMATION

Supplemental Information includes Supplemental Experimental Procedures and one figure and can be found with this article online at http://dx.doi.org/10.1016/j.celrep.2017.03.011.

AUTHOR CONTRIBUTIONS

A.W., J.R.W., P.J.M., and F.V.M. performed the studies. M.L. and M.B.-Y. generated the transgenic lines. A.W. and D.C. conceived and designed the experiments. All authors contributed to editing of the manuscript.

ACKNOWLEDGMENTS

This work was funded by the MRC UK. J.R.W. received an MRC/Imperial Chain-Florey Clinical PhD Fellowship. We thank Dr. Alex Sardini (MRC LMS) for help with the CLAMS studies and data analysis.

Received: August 15, 2016
Revised: February 2, 2017
Accepted: March 1, 2017
Published: March 28, 2017

REFERENCES

Arad, M., Seidman, C.E., and Seidman, J.G. (2007). AMP-activated protein kinase in the heart: role during health and disease. Circ. Res. *100*, 474–488.

Barnes, B.R., Marklund, S., Steiler, T.L., Walter, M., Hjälm, G., Amarger, V., Mahlapuu, M., Leng, Y., Johansson, C., Galuska, D., et al. (2004). The 5′-AMP-activated protein kinase gamma3 isoform has a key role in carbohydrate and lipid metabolism in glycolytic skeletal muscle. J. Biol. Chem. *279*, 38441–38447.

Barré, L., Richardson, C., Hirshman, M.F., Brozinick, J., Fiering, S., Kemp, B.E., Goodyear, L.J., and Witters, L.A. (2007). Genetic model for the chronic activation of skeletal muscle AMP-activated protein kinase leads to glycogen accumulation. Am. J. Physiol. Endocrinol. Metab. *292*, E802–E811.

Benziane, B., Björnholm, M., Lantier, L., Viollet, B., Zierath, J.R., and Chibalin, A.V. (2009). AMP-activated protein kinase activator A-769662 is an inhibitor of the Na(+)-K(+)-ATPase. Am. J. Physiol. Cell Physiol. *297*, C1554–C1566.

Bergeron, R., Previs, S.F., Cline, G.W., Perret, P., Russell, R.R., 3rd, Young, L.H., and Shulman, G.I. (2001). Effect of 5-aminoimidazole-4-carboxamide-1-beta-D-ribofuranoside infusion on in vivo glucose and lipid metabolism in lean and obese Zucker rats. Diabetes *50*, 1076–1082.

Carling, D., Thornton, C., Woods, A., and Sanders, M.J. (2012). AMP-activated protein kinase: new regulation, new roles? Biochem. J. *445*, 11–27.

Cool, B., Zinker, B., Chiou, W., Kifle, L., Cao, N., Perham, M., Dickinson, R., Adler, A., Gagne, G., Iyengar, R., et al. (2006). Identification and characterization of a small molecule AMPK activator that treats key components of type 2 diabetes and the metabolic syndrome. Cell Metab. *3*, 403–416.

Crute, B.E., Seefeld, K., Gamble, J., Kemp, B.E., and Witters, L.A. (1998). Functional domains of the alpha1 catalytic subunit of the AMP-activated protein kinase. J. Biol. Chem. *273*, 35347–35354.

Dandapani, M., and Hardie, D.G. (2013). AMPK: opposing the metabolic changes in both tumour cells and inflammatory cells? Biochem. Soc. Trans. *41*, 687–693.

Davies, S.P., Carling, D., Munday, M.R., and Hardie, D.G. (1992). Diurnal rhythm of phosphorylation of rat liver acetyl-CoA carboxylase by the AMP-activated protein kinase, demonstrated using freeze-clamping. Effects of high fat diets. Eur. J. Biochem. *203*, 615–623.

Donnelly, K.L., Smith, C.I., Schwarzenberg, S.J., Jessurun, J., Boldt, M.D., and Parks, E.J. (2005). Sources of fatty acids stored in liver and secreted via lipoproteins in patients with nonalcoholic fatty liver disease. J. Clin. Invest. *115*, 1343–1351.

Duarte, J.A., Carvalho, F., Pearson, M., Horton, J.D., Browning, J.D., Jones, J.G., and Burgess, S.C. (2014). A high-fat diet suppresses de novo lipogenesis and desaturation but not elongation and triglyceride synthesis in mice. J. Lipid Res. *55*, 2541–2553.

Dushay, J.R., Toschi, E., Mitten, E.K., Fisher, F.M., Herman, M.A., and Maratos-Flier, E. (2014). Fructose ingestion acutely stimulates circulating FGF21 levels in humans. Mol. Metab. *4*, 51–57.

Foretz, M., Ancellin, N., Andreelli, F., Saintillan, Y., Grondin, P., Kahn, A., Thorens, B., Vaulont, S., and Viollet, B. (2005). Short-term overexpression of a constitutively active form of AMP-activated protein kinase in the liver leads to mild hypoglycemia and fatty liver. Diabetes *54*, 1331–1339.

Foretz, M., Hébrard, S., Leclerc, J., Zarrinpashneh, E., Soty, M., Mithieux, G., Sakamoto, K., Andreelli, F., and Viollet, B. (2010). Metformin inhibits hepatic gluconeogenesis in mice independently of the LKB1/AMPK pathway via a decrease in hepatic energy state. J. Clin. Invest. *120*, 2355–2369.

Fullerton, M.D., Galic, S., Marcinko, K., Sikkema, S., Pulinilkunnil, T., Chen, Z.P., O'Neill, H.M., Ford, R.J., Palanivel, R., O'Brien, M., et al. (2013). Single phosphorylation sites in Acc1 and Acc2 regulate lipid homeostasis and the insulin-sensitizing effects of metformin. Nat. Med. *19*, 1649–1654.

Girard, J., Ferré, P., and Foufelle, F. (1997). Mechanisms by which carbohydrates regulate expression of genes for glycolytic and lipogenic enzymes. Annu. Rev. Nutr. *17*, 325–352.

Hardie, D.G., and Carling, D. (1997). The AMP-activated protein kinase–fuel gauge of the mammalian cell? Eur. J. Biochem. *246*, 259–273.

Hawley, S.A., Ford, R.J., Smith, B.K., Gowans, G.J., Mancini, S.J., Pitt, R.D., Day, E.A., Salt, I.P., Steinberg, G.R., and Hardie, D.G. (2016). The Na+/glucose cotransporter inhibitor canagliflozin activates AMPK by inhibiting mitochondrial function and increasing cellular AMP levels. Diabetes *65*, 2784–2794.

Henriksen, B.S., Curtis, M.E., Fillmore, N., Cardon, B.R., Thomson, D.M., and Hancock, C.R. (2013). The effects of chronic AMPK activation on hepatic triglyceride accumulation and glycerol 3-phosphate acyltransferase activity with high fat feeding. Diabetol. Metab. Syndr. *5*, 29.

Hudgins, L.C., Hellerstein, M.K., Seidman, C.E., Neese, R.A., Tremaroli, J.D., and Hirsch, J. (2000). Relationship between carbohydrate-induced hypertriglyceridemia and fatty acid synthesis in lean and obese subjects. J. Lipid Res. *41*, 595–604.

Matteoni, C.A., Younossi, Z.M., Gramlich, T., Boparai, N., Liu, Y.C., and McCullough, A.J. (1999). Nonalcoholic fatty liver disease: a spectrum of clinical and pathological severity. Gastroenterology *116*, 1413–1419.

Mayes, P.A. (1993). Intermediary metabolism of fructose. Am. J. Clin. Nutr. *58* (*5, Suppl*), 754S–765S.

Milan, D., Jeon, J.T., Looft, C., Amarger, V., Robic, A., Thelander, M., Rogel-Gaillard, C., Paul, S., Iannuccelli, N., Rask, L., et al. (2000). A mutation in PRKAG3 associated with excess glycogen content in pig skeletal muscle. Science *288*, 1248–1251.

Postic, C., and Girard, J. (2008). Contribution of de novo fatty acid synthesis to hepatic steatosis and insulin resistance: lessons from genetically engineered mice. J. Clin. Invest. *118*, 829–838.

Postic, C., Shiota, M., Niswender, K.D., Jetton, T.L., Chen, Y., Moates, J.M., Shelton, K.D., Lindner, J., Cherrington, A.D., and Magnuson, M.A. (1999). Dual roles for glucokinase in glucose homeostasis as determined by liver and pancreatic beta cell-specific gene knock-outs using Cre recombinase. J. Biol. Chem. *274*, 305–315.

Rui, L. (2014). Energy metabolism in the liver. Compr. Physiol. *4*, 177–197.

Schönke, M., Myers, M.G., Jr., Zierath, J.R., and Björnholm, M. (2015). Skeletal muscle AMP-activated protein kinase γ1(H151R) overexpression enhances whole body energy homeostasis and insulin sensitivity. Am. J. Physiol. Endocrinol. Metab. *309*, E679–E690.

She, P., Burgess, S.C., Shiota, M., Flakoll, P., Donahue, E.P., Malloy, C.R., Sherry, A.D., and Magnuson, M.A. (2003). Mechanisms by which liver-specific PEPCK knockout mice preserve euglycemia during starvation. Diabetes *52*, 1649–1654.

Stein, S.C., Woods, A., Jones, N.A., Davison, M.D., and Carling, D. (2000). The regulation of AMP-activated protein kinase by phosphorylation. Biochem. J. *345*, 437–443.

Steinberg, G.R., and Kemp, B.E. (2009). AMPK in health and disease. Physiol. Rev. *89*, 1025–1078.

Tappy, L., and Lê, K.A. (2010). Metabolic effects of fructose and the worldwide increase in obesity. Physiol. Rev. *90*, 23–46.

Viollet, B., Athea, Y., Mounier, R., Guigas, B., Zarrinpashneh, E., Horman, S., Lantier, L., Hebrard, S., Devin-Leclerc, J., Beauloye, C., et al. (2009). AMPK: Lessons from transgenic and knockout animals. Front. Biosci. (Landmark Ed.) *14*, 19–44.

Woods, A., Azzout-Marniche, D., Foretz, M., Stein, S.C., Lemarchand, P., Ferré, P., Foufelle, F., and Carling, D. (2000). Characterization of the role of AMP-activated protein kinase in the regulation of glucose-activated gene expression using constitutively active and dominant negative forms of the kinase. Mol. Cell. Biol. *20*, 6704–6711.

Xiao, B., Heath, R., Saiu, P., Leiper, F.C., Leone, P., Jing, C., Walker, P.A., Haire, L., Eccleston, J.F., Davis, C.T., et al. (2007). Structural basis for AMP binding to mammalian AMP-activated protein kinase. Nature *449*, 496–500.

Xiao, B., Sanders, M.J., Carmena, D., Bright, N.J., Haire, L.F., Underwood, E., Patel, B.R., Heath, R.B., Walker, P.A., Hallen, S., et al. (2013). Structural basis of AMPK regulation by small molecule activators. Nat. Commun. *4*, 3017.

Yavari, A., Stocker, C.J., Ghaffari, S., Wargent, E.T., Steeples, V., Czibik, G., Pinter, K., Bellahcene, M., Woods, A., Martínez de Morentin, P.B., et al. (2016). Chronic Activation of γ2 AMPK Induces Obesity and Reduces β Cell Function. Cell Metab. *23*, 821–836.

Younossi, Z.M., Koenig, A.B., Abdelatif, D., Fazel, Y., Henry, L., and Wymer, M. (2016). Global epidemiology of nonalcoholic fatty liver disease-Meta-analytic assessment of prevalence, incidence, and outcomes. Hepatology *64*, 73–84.

Zadra, G., Photopoulos, C., Tyekucheva, S., Heidari, P., Weng, Q.P., Fedele, G., Liu, H., Scaglia, N., Priolo, C., Sicinska, E., et al. (2014). A novel direct activator of AMPK inhibits prostate cancer growth by blocking lipogenesis. EMBO Mol. Med. *6*, 519–538.

Zhou, G., Myers, R., Li, Y., Chen, Y., Shen, X., Fenyk-Melody, J., Wu, M., Ventre, J., Doebber, T., Fujii, N., et al. (2001). Role of AMP-activated protein kinase in mechanism of metformin action. J. Clin. Invest. *108*, 1167–1174.

Zordoky, B.N.M., Nagendran, J., Pulinilkunnil, T., Kienesberger, P.C., Masson, G., Waller, T.J., Kemp, B.E., Steinberg, G.R., and Dyck, J.R.B. (2014). AMPK-dependent inhibitory phosphorylation of ACC is not essential for maintaining myocardial fatty acid oxidation. Circ. Res. *115*, 518–524.

Cell Reports, Volume *18*

Supplemental Information

Liver-Specific Activation of AMPK

Prevents Steatosis on a High-Fructose Diet

Angela Woods, Jennet R. Williams, Phillip J. Muckett, Faith V. Mayer, Maria Liljevald, Mohammad Bohlooly-Y, and David Carling

Figure S1. Related to Figure 4

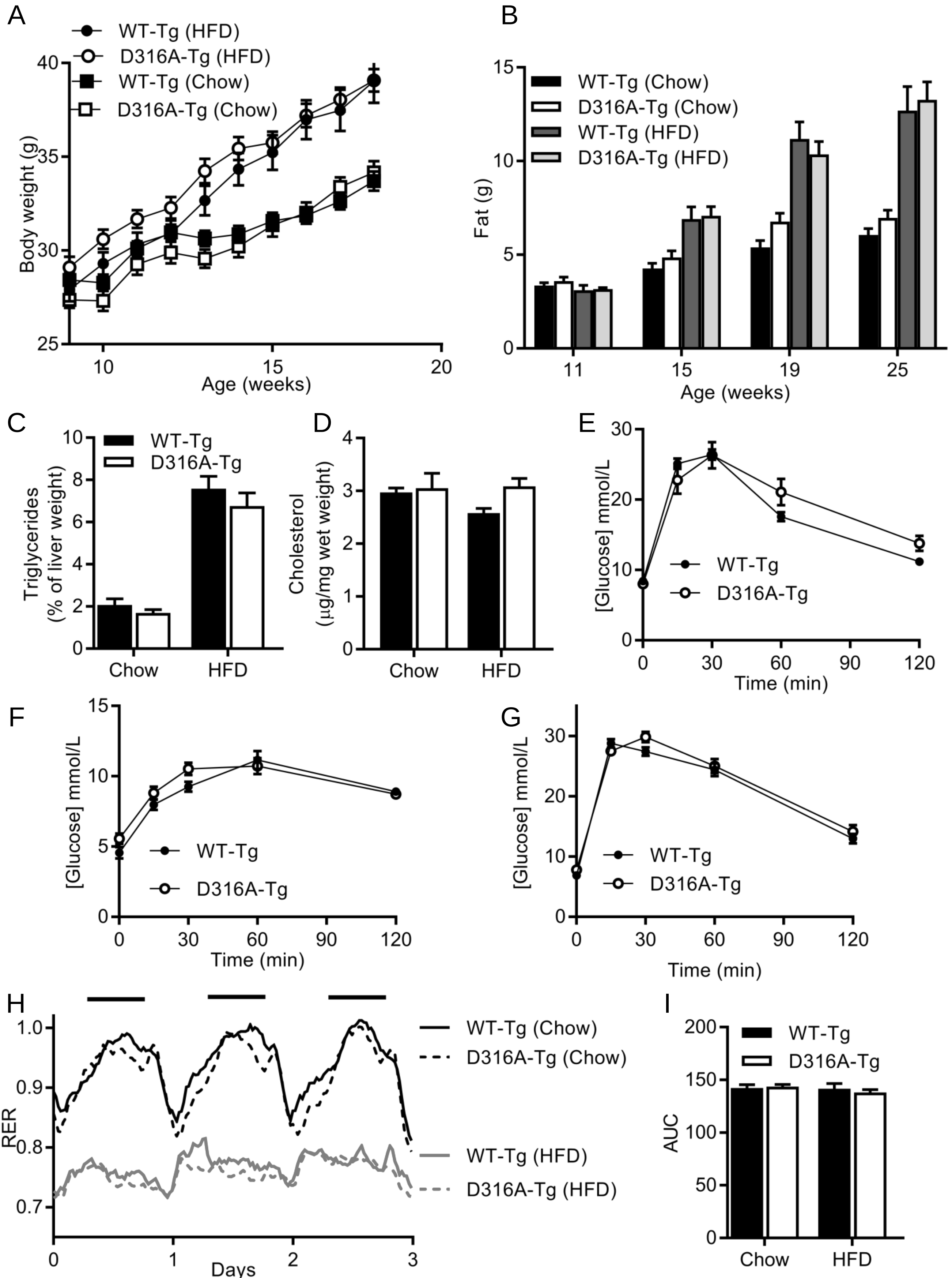

Figure S1. Metabolic effects of chronic hepatic activation of AMPK on mice fed a standard chow or high fat diet.

(A) Body weights of transgenic mice expressing either wild type γ1 AMPK (WT-Tg) or D316Aγ1 (D316A-Tg) fed a chow diet or a high fat diet (HFD) from 8 weeks of age. Results shown are the mean ± S.E.M. (in each case, n=24). (B) Total body fat, as assessed by EchoMRI (n=13-24). (C) Liver triglyceride and (D) liver cholesterol content (n= 9-12). (E) Glucose and (F) pyruvate tolerance tests of mice maintained on chow diet, and (G) glucose tolerance of mice fed a high fat diet for 12 weeks (n=9-10 per group). (H) Respiratory exchange ratio (RER) measured over 3 days for mice maintained on chow diet, or fed a high fat diet for 12 weeks (solid black bars represent hours of dark 19.00-7.00). (I) Total area under the curve (AUC) for VO_2 measured for mice maintained on chow diet, or fed a high fat diet for 12 weeks (n=14). In all cases results are shown as mean ± S.E.M. for the indicated number of mice.

Supplemental Experimental Procedures

Animal models

The *Rosa26* gene targeting vector was prepared from a mouse C57BL/6 bacterial artificial chromosome with homology arms 5.6 kb and 1.7 kb flanking the XbaI site in the *Rosa26* gene, described in detail previously (Soriano, 1999). A sequence encoding the Flag epitope (DYKDDDDK) at the C-terminus was engineered into the constructs to allow recognition by an anti-Flag antibody. Targeting vectors were linearized, and electroporated into PRXB6N mouse embryonic stem (ES) cells (Primogenix, Lauire, USA). Targeted ES cells were injected into BalbC/cANnCrl (Charles River, Germany) blastocysts and embryos were implanted into pseudo pregnant C57Bl6NCrl (Charles River, Germany) female mice. The resulting chimeric animals were mated with C57BL/6N mice (Charles River, Germany) to produce agouti heterozygous animals (F1). To generate animals without the Neo cassette, F1 mice were bred with CAG-FlpO. For genotyping the following primers were used: P1:MLi713F, accatgttcatgccttcttctt end of CAG promoter, P2=MLi748R, aattaggtcatagcagcgatga start of cDNA, detecting a fragment of 1.1 kb. These mice were crossed with *B6.Cg-Tg (Alb-Cre) 21 Mgn/J* transgenic mice harbouring Cre-recombinase under the albumin promoter (stock number 003574, Jackson Laboratories, Maine USA). Mice were maintained on a 12 h light/dark cycle with free access to food and water and housed in specific-pathogen free barrier facilities. Animals were killed by cervical dislocation and organs harvested rapidly and frozen in liquid nitrogen.

Primary mouse hepatocytes

Hepatocytes were isolated by collagenase perfusion of the liver of anesthetised mice. After isolation cells were seeded in collagen coated dishes in Medium 199 with Earles and L-glutamine (Gibco) supplemented with UltroserG (Pall Life Sciences), 1% albumin, 100 nM insulin, 100 nM triiodothyronine (T_3) and 100 nM dexamethasone. After cell attachment, the hepatocytes were cultured for 16–18 h in the absence of T3, albumin and UltroserG and in the presence of 1 nM insulin.

Hepatic Glucose Output

Cells were transferred into Dulbecco's Modified Eagles media without phenol red and supplemented with 2 mM sodium pyruvate and 20 mM lactate. Aliquots of media were removed from the cells and the glucose concentration measured using a glucose oxidase kit (Sigma).

Lipogenesis

Hepatocytes were incubated in M199 media containing 2 mM sodium acetate and 3 µCi/ml [1-^{14}C]sodium acetate (Perkin Elmer) for 4 h. Cells were washed thoroughly then scraped into methanol. Methanol was evaporated off and the pellet extracted with methyl-tert butyl ether:methanol (3:1), insoluble material was removed by centrifugation and the soluble fraction evaporated to dryness before determining ^{14}C incorporation by scintillation counting.

Fatty acid oxidation

Hepatocytes were incubated in M199 media containing 0.5 mM palmitate conjugated to BSA (2% w/v) and 1 µCi/ml [1-^{14}C]palmitic acid (Perkin Elmer). After 3-6 h media was removed and acidified with 0.1 volume 17 N acetic acid in an air-tight vial and the resulting CO_2 released was trapped in filter paper soaked in 1 M NaOH. The ^{14}C dissolved in NaOH was quantified by scintillation counting. Cells were washed thoroughly in PBS, extracted with chloroform/methanol and ^{14}C incorporated into aqueous soluble intermediates was determined. Total oxidation was calculated as the sum of $^{14}CO_2$-produced and ^{14}C incorporated into incomplete oxidation products.

Quantitative RT-PCR analysis

RNA was isolated from livers by homogenization in Trizol reagent (Invitrogen) according to the manufacturer's instructions, followed by purification on an RNeasy column (Qiagen). First strand cDNA synthesis using Superscript II (Invitrogen) according to the manufacturer's instructions and quantitative PCR was done with SensiMix Plus SYBR kit (Quantace) using the following primers:

G6Pase: CGACTCGCTATCTCCAAGTGA, GTTGAACCAGTCTCCGACCA

PEPCK: CCCCTTGTCTATGAAGCCCTCA, GCCCTTGTGTTCTGCAGCAG

ACC1: ATGGGCGGAATGGTCTCTTTC, TGGGGACCTTGTCTTCATCAT

FAS: TGCTCCCAGCTGCAGGC, GCCCGGTAGCTCTGGGTGTA

SREBP1c: GGAGCCATGGATTGCACATT, GCTTCCAGAGAGGAGGCCAG

SPOT14: Primers were obtained from Qiagen (no sequence information provided).

AMPK assay

AMPK was immunoprecipitated from liver or hepatocyte cell lysates using either a rabbit pan-β antibody bound to protein A-Sepharose (Woods et al., 1996) or anti-Flag affinity resin (Sigma) and activity present in the immune complexes was determined by phosphorylation of the SAMS peptide, as previously described (Davies et al., 1989).

Western blotting

Proteins were resolved by SDS-PAGE (Novex bis-tris 4-12% gradient gels) and transferred to polyvinylidene difluoride membrane. Primary antibodies used were: actin (Sigma), FAS (BD laboratories), FGF21 (Sigma), PP2Cα (Acris Antibodies), SCD1 (Life Technology), tubulin (Sigma). ACC, pACC (S79), AMPKα1/α2, AMPKβ1/β2, pAMPK (T172) and Flag were all from Cell Signaling Technology. Primary antibodies were detected using LI-COR IRDye® Infrared Dye secondary antibodies and visualised using an Odyssey Infrared Imager (LI-COR Biotechnology). Quantification of results was performed using Odyssey software and in some cases expressed as a ratio of the signal obtained with the phospho-specific antibody relative to an appropriate loading control antibody.

Recombinant AMPK expression

AMPK complexes were expressed in *E.coli* BL21 (DE3) cells, purified by affinity chromatography using nickel-Sepharose and phosphorylated by incubation with CaMKKβ as described previously (Sanders et al., 2007). Dephosphorylation of AMPK by recombinant PP2Cα was monitored either by Western blotting of phospho-T172 or by assaying for AMPK activity using the phosphorylation of the SAMS peptide. AMP present in perchloric acid extracts of purified AMPK was determined by ion-exchange chromatography as previously described (Fryer et al., 2002). Prior to extraction with perchloric acid, ADP (4 nmol) was added to control for efficiency of the extraction process.

Metabolic parameters

In vivo oxygen consumption and carbon dioxide production were measured using a Columbus Instruments Comprehensive Lab Animal Monitoring System over a 3 day period and used to calculate respiratory exchange ratio. Serum levels of total cholesterol, LDL and HDL cholesterol, triglycerides, 3-hydroxybutyrate and liver transaminases were determined by the Mouse Biochemistry Laboratories, Cambridge, UK. Lipids were extracted from tissue by homogenisation in ethanol (0.03 x w/v). Triglyceride content was measured using Triglyceride liquid (Sentinel Diagnostics) and cholesterol measured using Amplex Red Cholesterol kit (Molecular Probes). Liver glycogen was determined by alkaline hydrolysis by incubating tissue in 30% KOH at 100°C for 1h. Glycogen was precipitated in the cold by addition of 10x volume of ethanol and 7 μM LiCl. Precipitated glycogen was collected by centrifugation and dissolved in 4 M HCl, incubated for 1h at 100°C and neutralised by addition of an equal volume of K_2CO_3. The amount of glycogen converted to glucose was determined using a glucose oxidase kit (Sigma). Glucose and pyruvate tolerance tests were performed on mice after a 16 hour overnight fast. Animals were injected intraperitoneally with glucose (2 g/kg or 1.5 g/kg for mice on high fat diet) or pyruvate (2 g/kg) and blood glucose levels determined by a glucometer at the indicated time points. Body composition for determining body fat was measured by MRI using an EchoMRI-100 (Echo Medical Systems).

Supplemental References

Davies, S.P., Carling, D., and Hardie, D.G. (1989). Tissue distribution of AMP-activated protein kinase, and lack of activation by cyclic AMP-dependent protein kinase, studied using a specific and sensitive peptide assay. Eur. J. Biochem. *186*, 123-128.

Fryer, L.G., Parbu-Patel, A., and Carling, D. (2002). The Anti-diabetic drugs rosiglitazone and metformin stimulate AMP-activated protein kinase through distinct signaling pathways. J. Biol. Chem. *277*, 25226-25232.

Sanders, M.J., Grondin, P.O., Hegarty, B.D., Snowden, M.A., and Carling, D. (2007). Investigating the mechanism for AMP activation of the AMP-activated protein kinase cascade. Biochem. J. *403*, 139-148.

Soriano, P. (1999). Generalized lacZ expression with the ROSA26 Cre reporter strain Nat. Genet. *21*, 70-71.

Woods, A., Cheung, P.C., Smith, F.C., Davison, M.D., Scott, J., Beri, R.K., and Carling, D. (1996). Characterization of AMP-activated protein kinase beta and gamma subunits. Assembly of the heterotrimeric complex in vitro. J. Biol. Chem. *271*, 10282-10290.
